# Patients with Single-Ventricle Physiology over the Age of 40 Years

**DOI:** 10.3390/jcm9124085

**Published:** 2020-12-18

**Authors:** Claudia Pujol, Sandra Schiele, Susanne J. Maurer, Julia Hock, Celina Fritz, Alfred Hager, Peter Ewert, Oktay Tutarel

**Affiliations:** 1 Department of Congenital Heart Disease and Paediatric Cardiology, German Heart Centre Munich, Technical University of Munich, 80636 Munich, Germany; claupujol@gmail.com (C.P.); schiele@dhm.mhn.de (S.S.); julia.hock@tum.de (J.H.); celina.fritz88@googlemail.com (C.F.); hager@dhm.mhn.de (A.H.); ewert@dhm.mhn.de (P.E.); 2 Department of Electrophysiology, German Heart Centre Munich, Technical University of Munich, 80636 Munich, Germany; susanne.maurer@tum.de; 3 DZHK (German Centre for Cardiovascular Research), Partner Site Munich Heart Alliance, 80992 Munich, Germany

**Keywords:** single-ventricle physiology, mortality, renal disease, adult congenital heart disease, Fontan

## Abstract

Background: Single-ventricle physiology (SVP) is associated with significant morbidity and mortality at a young age. However, survival prospects have improved and risk factors for a negative outcome are well described in younger cohorts. Data regarding older adults is scarce. Methods: In this study, SVP patients under active follow-up at our center who were ≥40 years of age at any point between January 2005 and December 2018 were included. Demographic data, as well as medical/surgical history were retrieved from hospital records. The primary end-point was all-cause mortality. Results: Altogether, 49 patients (19 female (38.8%), mean age 49.2 ± 6.4 years) were included. Median follow-up time was 4.9 years (interquartile range (IQR): 1.8–8.5). Of these patients, 40 (81.6%) had undergone at least one cardiac surgery. The most common extracardiac comorbidities were thyroid dysfunction (*n* = 27, 55.1%) and renal disease (*n* = 15, 30.6%). During follow-up, 10 patients (20.4%) died. On univariate analysis, renal disease and liver cirrhosis were predictors of all-cause mortality. On multivariate analysis, only renal disease (hazard ratio (HR): 12.5, 95% confidence interval (CI): 1.5–106.3, *p* = 0.021) remained as an independent predictor. Conclusions: SVP patients ≥40 years of age are burdened with significant morbidity and mortality. Renal disease is an independent predictor of all-cause mortality.

## 1. Introduction

Life expectancy for those born with congenital heart disease (CHD) has increased over the last few decades [1]. Accordingly, the number of adults with congenital heart disease (ACHD) is increasing and patients are reaching older age [2]; the median age of ACHD patients has increased to 40 years [1]. This is the case for patients with simple defects, as well as those with complex defects [3]. CHDs with single-ventricle physiology (SVP) are one of the most complex, with only a guarded life expectancy if left untreated. In the 1970s, the concept of the Fontan operation was introduced, which dramatically improved the outcome for these patients [4]. In one large series, overall, 10-, 20-, and 30-year survival rates were 74%, 61%, and 43%, respectively [4]. Still, sequelae like arrhythmias, thromboembolic events and premature deaths in the first decades of life have been reported [4]. While the outcome in these younger patients with SVP has been extensively studied, data regarding older patients (above 40 years of age) are scarce [5]. This is also true for patients with SVP who have not had the Fontan operation [6,7].

Therefore, the aim of this study is to examine the clinical course of patients with SVP over the age of 40 years and assess the predictors of a worse outcome.

## 2. Materials and Methods

This retrospective, single-center study included all patients with a diagnosis of SVP under follow-up at a tertiary ACHD center who were ≥40 years of age at any point between January 2005 and March 2018. The time-point of inclusion and begin of follow-up was either the date of their 40th birthday or, if the patient was already 40 years old in the year 2005, the first visit after the 1st of January 2005.

The primary end-point was all-cause mortality. Demographic data and information on medical/surgical history were retrieved from hospital records. Symptomatic status was assessed according to the New York Heart Association (NYHA) classification.

The underlying CHD was classified as follows: double inlet left ventricle (DILV), tricuspid atresia (TA), double outlet right ventricle (DORV), and other.

Based on the results of routine transthoracic echocardiograms, systemic ventricular systolic function was graded semi-quantitatively as normal, mildly impaired, moderately impaired or severely impaired, as described previously [8].

Arrhythmias encompassed any type of supraventricular or ventricular arrhythmia requiring therapy. Coronary artery disease was present if proven by coronary angiogram or with a history of percutaneous coronary intervention or aortocoronary bypass surgery. Cyanosis was defined as a resting oxygen saturation below 90%. Clinical practice guidelines criteria were used to diagnose renal disease (structural or functional kidney damage ≥3 months or glomerular filtration rate (GFR) ≤ 60 mL/min/1.73 m^2^ ≥ 3 months) and pulmonary hypertension [9,10]. Lung disease included any form of it (i.e., asthma, chronic obstructive lung disease, emphysema, etc.). Patients with diabetes included both insulin-dependent and non-insulin-dependent cases. Heart failure was diagnosed according to recent guidelines [11].

Statistical analyses were performed using SPSS version 25 (IBM Corp, Armonk, NY, USA) and MedCalc version 19.0.3.0 (MedCalc Software, Mariakerke, Belgium). Continuous variables are presented as mean ± standard deviation (SD) or median (interquartile range (IQR)), whereas categorical variables are presented as number (percentage). Comparisons between groups were performed using the Mann–Whitney U test or Student’s *t*-test for continuous and Chi-square test for categorical variables. Univariate Cox proportional hazards analysis was used to assess the association between variables and the primary endpoint. Significant variables (*p* < 0.05) were subsequently included in a multivariable Cox proportional hazards analysis model. All tests were performed two-sided and for all analyses, a *p*-value < 0.05 was considered statistically significant.

This study complies with the Declaration of Helsinki, and the ethics committee of the Medical Faculty of the Technical University of Munich has approved the research protocol (124/18 S). The requirement for informed consent was waived by the ethics committee due to the retrospective nature of the study.

## 3. Results

Altogether, 49 patients (19 female (38.8%), mean age 49.2 ± 6.4 years) were included. Median follow-up time was 4.9 years (IQR: 1.8–8.5). Two patients had no follow-up visit. The underlying CHD was a DILV in 23 patients (46.9%), TA in 20 (40.8%), and miscellaneous in six (12.2%: DORV *n* = 1, undetermined *n* = 4, criss-cross heart *n* = 1). More detailed information regarding baseline characteristics can be found in Table 1.

Regarding symptoms, 11 patients (22.4%) were in NYHA class I, 29 (59.2%) in NYHA class II, and 9 (18.4%) in NYHA class III. At the end of follow-up, only six patients (12.2%) remained in NYHA class I, while 25 (51%) were in NYHA class II, 13 (26.5%) in NYHA class III, and three (6.1%) in NYHA class IV (*p* = 0.001).

Out of the 49 patients, 40 (81.6%) had undergone at least one cardiac surgery. Nine patients never underwent cardiac surgery. In the patients who underwent surgery, a variation of the Fontan operation was present in 31 (77.5%): atriopulmonary Fontan in 20, and a total cavo-pulmonary connection (TCPC) in 11 (five of these were converted to a TCPC from a previous atriopulmonary Fontan). Mean age at conversion was 37.8 ± 8.6 years (median: 39 years, range: 25–48). Indications for conversion surgery were arrhythmia and Fontan-failure (protein losing-enteropathy). All patients survived the surgery, but one patient died two years later due to heart failure. Three patients (6.1%) underwent cardiac surgery during follow-up: two TCPC conversions and one patch refixation in an atriopulmonary Fontan.

In the group of patients who underwent cardiac surgery without a Fontan palliation, two had cavo-pulmonary shunts (one Glenn shunt, one partial cavo-pulmonary connection), three Blalock–Taussig shunts (BT), and one had a BT shunt with a Glenn shunt. Three patients had operations without shunts: one patient with a right ventricle to pulmonary artery conduit, one patient with pulmonary artery banding, and one patient with mitral valve repair. Restrictive pulmonary flow was present in 10 out of these 20 patients (six unoperated); two patients had Eisenmenger syndrome, neither of which had cardiac surgery.

During follow-up, 19 patients (38.8%) underwent cardiac catheterizations, and three of them had more than one catheterization. Altogether, 19 cardiac catheterizations were performed: 15 were diagnostic and four were interventional (two Melody valve implantations in the atrioventricular valve position, one dilatation of the pulmonary artery, one closure of collateral veins).

Arrhythmias occurred frequently. At baseline, 17 patients (34.7%) already had at least one electrophysiological procedure (EP), while during follow-up, nine patients (18.4%) had an EP. Out of these, five had more than one procedure. Treatment with cardioversion was necessary in 20 patients (40.8%); in 14 of these, multiple cardioversions were necessary. A pacemaker was present in 13 patients (26.5%) at baseline, while eight (16.3%) needed a pacemaker during follow-up.

During follow-up, 10 patients (20.4%) died. The cause of mortality was cardiovascular in six patients (60%), non-cardiovascular in two (20%; sepsis secondary to pneumonia, liver failure) and unknown in two (20%).

**Figure 1:**
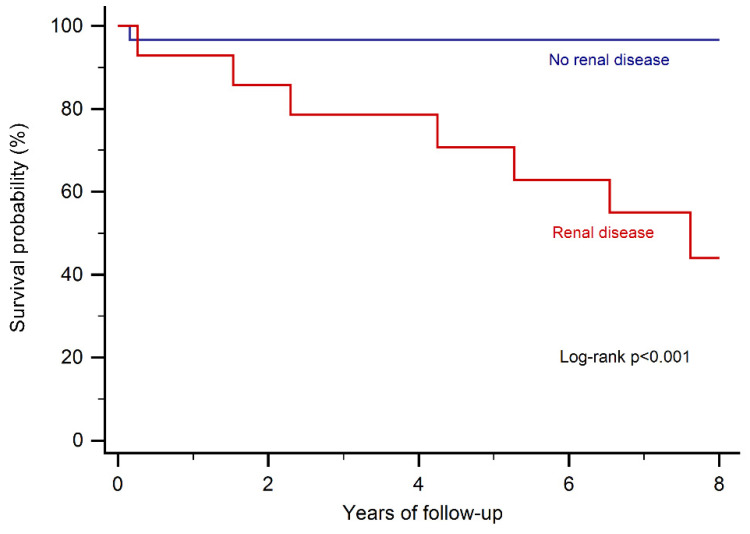
Kaplan–Meier curves stratifying patients according to the presence of renal disease.

On univariate analysis, renal disease (hazard ratio (HR): 17.6, 95% CI: 2.2–140.1, *p* < 0.01) and liver cirrhosis (HR: 5.7, 95% CI: 1.6–20.2, *p* < 0.01) were predictors of all-cause mortality. On multivariate analysis, only renal disease (HR: 12.5, 95% CI: 1.5–106.3, *p* = 0.021) remained as an independent predictor (Figure 1).

The most common comorbidities at baseline were thyroid dysfunction (*n* = 27 (55.1%), out of these hypothyroidism *n* = 25, hyperthyroidism *n* = 2) and renal disease (*n* = 15 (30.6%), (Table 2). At baseline, five patients (10.2%) had a protein losing enteropathy, two patients (4.1%) had a history of infective endocarditis, 13 patients (26.5%) had a history of a cerebrovascular accident (CVA), four patients (8.1%) had a history of an abscess (three brain and one lung), two patients (3.9%) had a history of systemic emboli (4.1%) and nine patients (18.4%) had a history of venous thrombosis (four deep vein thrombosis, four atrial thrombi and one valve thrombosis). Altogether, 49 admissions for heart failure in 35 patients were recorded.

The following events occurred during the follow-up period: infective endocarditis in one patient (2%), CVA in six patients (12.2%), abscess in one patient (2%), systemic emboli in four patients (8.1%) and venous thrombosis in three patients (6.1%), Heart failure admissions occurred in 19 patients (38.8%), and ventricular tachycardia in three patients (6.1%). None of the patients were referred for heart transplantation or heart–lung transplantation.

## 4. Discussion

Patients with SVP over the age of 40 years were burdened with significant morbidity and mortality in this study. While extracardiac comorbidities were common, the presence of renal disease was an independent predictor of all-cause mortality.

Around 20% of the patients in our cohort died during a median follow-up of 4.9 years. This adds to the picture of 83% survival 25 years after Fontan completion reported from a large registry in Australia and New Zealand [12]. Considering that the patients in this registry were around five years of age at the time of Fontan completion, this translates into a mortality of 17% until the age of 30 years [12]. A study from the Mayo Clinic pointed to the different survival prospects according to the operative era [4]. For the era 1973–1990, corresponding to the era in which our patients underwent surgery, the 30-year survival rate was only 39% [4]. Our data complement these results, because they add information towards what to expect in these survivors who reach the age of 40 years.

Similar to previous studies in younger cohorts [12], the main cause of death in our cohort was cardiovascular; and the only independent predictor of mortality was renal disease. This is in accordance with previous results from cohorts of ACHD patients with varying underlying CHD. Dimopoulos et al. demonstrated in a large cohort of 1102 ACHD patients with a mean age of 36 years, a three-fold higher mortality than normal in patients with moderate to severe renal dysfunction [13]. In a recent study in ACHD patients over the age of 40 years with a variability of underlying CHD, kidney dysfunction was a potent predictor of one-year mortality risk after an incident heart failure hospitalization [14]. The cause of renal disease in SVP patients is probably multifactorial, including elevated venous pressures, relatively low cardiac output and prior episodes of peri-operative acute kidney injury [15]. While renal disease is quite common in SVP, especially in patients after the Fontan operation, it appears to be tolerated well until early adulthood. However, the authors of a recent review speculated, “… it is possible that unfavorable outcomes may be seen only with longer follow-up…” [15]. This was the case in our cohort of older adults with SVP, in which renal disease was an important predictor of mortality.

Liver cirrhosis was a univariate predictor of all-cause mortality in our study but did not remain in the final model. Liver cirrhosis is common in patients with SVP. In a large series of Fontan patients from the Mayo Clinic, 10-, 20-, and 30-year freedom from cirrhosis was 99%, 94%, and 57% [16]. Liver disease is already present in pediatric patients with Fontan physiology [17]. The pathophysiology is multifactorial and includes factors like central venous hypertension/passive congestion, as well as hypoxia due to systemic ventricular dysfunction and collaterals [18]. Routine liver health surveillance in this population should be implemented, especially considering the risk of developing hepatocellular carcinoma, but treatment options remain limited [17,19]. Liver transplantation or combined heart–liver transplantation could be an option in selected cases.

A recent study in a large cohort of adult congenital heart disease patients with heart failure reported brain natriuretic peptide (BNP) and sodium levels as predictors of a combined primary outcome including death, transplant, or ventricular assist device implantation [20]. These laboratory values were not available in all our patients and were therefore not included in the Cox proportional hazards analysis. However, even in the mentioned study, BNP was less predictive in SVP and cyanotic patients [20].

Functional status declined during the follow-up in our cohort, as was described for younger cohorts with a Fontan operation [21]. It is well known that patients with SVP have a reduced exercise capacity [22], which is associated with increased morbidity [23]. Therefore, interventions to improve the functional status and exercise capacity of these patients are needed. Exercise training seems to be safe and beneficial in these patients, but data for older patients is scarce [24].

During follow-up, a significant number of interventions, especially for arrhythmias, was necessary in our cohort. This finding was not unexpected. Atrial tachyarrhythmia is prevalent and associated with substantial morbidity in these patients [25]. Furthermore, their prevalence increases with follow-up duration [25]. Nonetheless, our results emphasize the burden of morbidity caused by arrhythmias in these patients, as well as the increasing demand on healthcare systems.

A limitation of our study is its retrospective nature with all its obvious drawbacks. In some patients, variables like the cause of death were missing. Furthermore, while the study may inform us about the current cohort of SVP patients over the age of 40 years, future cohorts might be different with improved surgical outcomes as a result of the widespread adaptation of the TCPC procedure on the one hand, and more complex patients like those with hypoplastic left heart syndrome on the other.

## 5. Conclusions

Patients with SVP over the age of 40 years are burdened with significant morbidity and mortality. Renal disease was an independent predictor of all-cause mortality.

## Figures and Tables

**Table 1:** Baseline characteristics of the study population.

	All	Alive	Dead	*p*
*n*	49 (100.0)	39 (79.6)	10 (20.4)	
Age (years)	49.2 ± 6.4	49.6 ± 6.9	47.8 ± 3.6	0.61
Female, *n* (%)	19 (38.8)	14 (35.8)	5 (50.0)	0.41
Congenital heart defect, *n* (%)				0.1
Double-inlet left ventricle	23 (46.9)	21 (53.8)	2 (20.0)	
Tricuspid atresia	20 (40.8)	13 (33.3)	7 (70.0)	
Miscellaneous	6 (12.2)	5 (12.8)	1 (10.0)	
Type of Fontan, *n* (%)				0.94
Atriopulmonal	20 (40.8)	15 (38.4)	5 (50.0)	
TCPC	11 (22.4)	9 (23.1)	2 (20.0)	
Other surgical correction	9 (18.4)	7 (17.9)	2 (20.0)	
Native	9 (18.4)	8 (20.5)	1 (10.0)	
Ventricle morphology, *n* (%)				0.81
Left	45 (91.8)	36 (92.3)	9 (90.0)	
Right	4 (8.1)	3 (7.7)	1 (10.0)	
Rhythm, *n* (%)				0.41
Sinus rhythm	23 (46.9)	18 (46.2)	5 (50)	
Atrial fibrillation	8 (16.3)	7 (17.9)	1 (10)	
Pacing rhythm	13 (26.5)	11 (28.2)	2 (20)	
Junctional rhythm	1 (2)	1 (2.6)	0	
Atrial flutter	1 (2)	1 (2.6)	0	
Other	3 (6.1)	1 (2.6)	2 (20)	
Pacemaker, *n* (%)	13 (26.5)	11 (28.2)	2 (20)	0.64
Prior heart failure admissions, *n* (%)	35 (71.4)	25 (64.1)	10 (100)	0.044
Cyanosis, *n* (%)	27 (55.1)	22 (56.4)	5 (50.0)	0.71
Hemoglobin, g/dL	17.0 ± 2.9	17.1 ± 2.8	16.8 ± 3.6	0.92
NT-pro BNP, ng/L *	1641 ± 2735	1606 ± 2981	1774 ± 1629	0.16
Cardiac medication, *n* (%)				
ACE-inhibitors/AT-Blocker	8 (16.3)	7 (17.9)	1 (10.0)	0.58
Beta-blocker	34 (69.4)	27 (69.2)	7 (70)	0.92
MRA	30 (61.2)	21 (53.8)	9 (90)	0.04
Digoxin	14 (28.6)	10 (25.6)	4 (40.0)	0.37
Diuretics	36 (73.4)	27 (69.2)	9 (90.0)	0.18
Anticoagulation, *n* (%)				0.75
None	10 (20.4)	10 (24.4)	1 (10.0)	
Vitamin K antagonist	36 (73.5)	27 (69.2)	9 (90.0)	
Direct oral anticoagulants	1 (2.0)	1 (2.6)	0	
Acetylsalicylic acid	1 (2.0)	1 (2.6)	0	
Acetylsalicylic acid + Clopidogrel	1 (2.0)	1 (2.6)	0	
Advanced PAH therapies	8 (16.3)	3 (7.7)	5 (50%)	0.01
NYHA class, *n* (%)				0.16
I	11 (22.4)	11 (28.2)	0	
II	29 (59.2)	23 (56.4)	7 (70.0)	
III	9 (18.4)	6 (15.4)	3 (30.0)	
Ventricular function, *n* (%)				0.97
Normal	11 (22.4)	9 (23.1)	2 (20.0)	
Mild	17 (34.7)	14 (35.9)	3 30.0)	
Moderate	17 (34.6)	13 (33.3)	4 (40.0)	
Severe	4 (8.2)	3 (7.7)	1 (10.0)	
Systemic AV valve regurgitation, *n* (%)				0.61
None	6 (12.2)	4 (10.3)	2 (20)	
Mild	26 (53%)	20 (51.3)	6 (60)	
Moderate	14 (28.6)	12 (30.7)	2 (20)	
Severe	3 (6.1)	3 (7.7)	0	

ACE: angiotensin-converting enzyme; AT: angiotensin II type I receptor; AV: atrioventricular; NYHA: New York Heart Association; NT-proBNP: N-terminal pro–B-type natriuretic peptide; MRA: mineralocorticoid/aldosterone receptor antagonists; PAH: pulmonary arterial hypertension; TCPC: total cavo-pulmonary connection. *p* value is for comparison between patients who died and those that are alive (Mann–Whitney U test was used for continuous and Chi-square test for categorical variables). *: available in 38 patients.

**Table 2:** Comorbidities at baseline, *n* (%).

	All	Alive	Dead	*p*
*n*	49 (100.0)	39 (79.6)	10 (20.4)	
Arterial hypertension	4 (8.2)	3 (7.7)	1 (10.0)	0.81
Hypercholesterolemia	5 (10.2)	5 (12.8)	0	0.23
Diabetes	5 (10.2)	5 (12.8)	0	0.23
Cancer	4 (8.2)	2 (5.1)	2 (20.0)	0.13
Depression	8 (16.3)	6 (15.4)	2 (20.0)	0.73
Endocarditis	2 (2)	2 (2.5)	0	0.47
Abscess	4 (8.2)	3 (7.7)	1 (10.0)	0.73
CVA	13 (26.5)	10 (25.6)	3 (30.0)	0.78
Thromboembolic events	9 (18.4)	6 (15.4)	3 (30.0)	0.29
Systemic embolism *	2 (4.0)	2 (5.1)	0	0.47
Thyroid dysfunction	27 (55.1)	20 (51.3)	7 (70.0)	0.29
Liver cirrhosis	8 (16.3)	3 (7.7)	5 (50.0)	0.001
Renal disease	15 (30.6)	6 (15.4)	9 (90.0)	<0.0001
Protein losing enteropathy	5 (10.2)	3 (7.7)	2 (20.0)	0.25

CVA: cerebrovascular accident. * other than CVA.

## References

[1] Lui G.K., Saidi A., Bhatt A.B., Burchill L.J., Deen J.F., Earing M.G., Gewitz M., Ginns J., Kay J.D., Kim Y.Y. (2017). Diagnosis and Management of Noncardiac Complications in Adults With Congenital Heart Disease: A Scientific Statement From the American Heart Association. Circulation.

[2] Hock J., Schwall L., Pujol C., Hager A., Oberhoffer R., Ewert P., Tutarel O. (2020). Tetralogy of Fallot or Pulmonary Atresia with Ventricular Septal Defect after the Age of 40 Years: A Single Center Study. J. Clin. Med..

[3] Tutarel O., Kempny A., Alonso-Gonzalez R., Jabbour R., Li W., Uebing A., Dimopoulos K., Swan L., Gatzoulis M.A., Diller G.P. (2014). Congenital heart disease beyond the age of 60: Emergence of a new population with high resource utilization, high morbidity, and high mortality. Eur. Heart J..

[4] Pundi K.N., Johnson J.N., Dearani J.A., Pundi K.N., Li Z., Hinck C.A., Dahl S.H., Cannon B.C., O’Leary P.W., Driscoll D.J. (2015). 40-Year Follow-Up After the Fontan Operation: Long-Term Outcomes of 1,052 Patients. J. Am. Coll. Cardiol..

[5] Motoki N., Ohuchi H., Miyazaki A., Yamada O. (2009). Clinical profiles of adult patients with single ventricular physiology. Circ. J..

[6] Gatzoulis M.A., Munk M.D., Williams W.G., Webb G.D. (2000). Definitive palliation with cavopulmonary or aortopulmonary shunts for adults with single ventricle physiology. Heart.

[7] Poterucha J.T., Anavekar N.S., Egbe A.C., Julsrud P.R., Connolly H.M., Ammash N.M., Warnes C.A. (2016). Survival and outcomes of patients with unoperated single ventricle. Heart.

[8] Bolger A.P., Sharma R., Li W., Leenarts M., Kalra P.R., Kemp M., Coats A.J.S., Anker S.D., Gatzoulis M.A. (2002). Neurohormonal Activation and the Chronic Heart Failure Syndrome in Adults With Congenital Heart Disease. Circulation.

[9] National Kidney Foundation (2002). K/DOQI clinical practice guidelines for chronic kidney disease: Evaluation, classification, and stratification. Am. J. Kidney Dis..

[10] Galie N., Humbert M., Vachiery J.L., Gibbs S., Lang I., Torbicki A., Simonneau G., Peacock A., Vonk Noordegraaf A., Beghetti M. (2016). 2015 ESC/ERS Guidelines for the diagnosis and treatment of pulmonary hypertension: The Joint Task Force for the Diagnosis and Treatment of Pulmonary Hypertension of the European Society of Cardiology (ESC) and the European Respiratory Society (ERS): Endorsed by: Association for European Paediatric and Congenital Cardiology (AEPC), International Society for Heart and Lung Transplantation (ISHLT). Eur. Heart J..

[11] Budts W., Roos-Hesselink J., Radle-Hurst T., Eicken A., McDonagh T.A., Lambrinou E., Crespo-Leiro M.G., Walker F., Frogoudaki A.A. (2016). Treatment of heart failure in adult congenital heart disease: A position paper of the Working Group of Grown-Up Congenital Heart Disease and the Heart Failure Association of the European Society of Cardiology. Eur. Heart J..

[12] d’Udekem Y., Iyengar A.J., Galati J.C., Forsdick V., Weintraub R.G., Wheaton G.R., Bullock A., Justo R.N., Grigg L.E., Sholler G.F. (2014). Redefining expectations of long-term survival after the Fontan procedure: Twenty-five years of follow-up from the entire population of Australia and New Zealand. Circulation.

[13] Dimopoulos K., Diller G.P., Koltsida E., Pijuan-Domenech A., Papadopoulou S.A., Babu-Narayan S.V., Salukhe T.V., Piepoli M.F., Poole-Wilson P.A., Best N. (2008). Prevalence, predictors, and prognostic value of renal dysfunction in adults with congenital heart disease. Circulation.

[14] Wang F., Liu A., Brophy J.M., Cohen S., Abrahamowicz M., Paradis G., Marelli A. (2020). Determinants of Survival in Older Adults With Congenital Heart Disease Newly Hospitalized for Heart Failure. Circ. Heart Fail..

[15] Zafar F., Lubert A.M., Katz D.A., Hill G.D., Opotowsky A.R., Alten J.A., Goldstein S.L., Alsaied T. (2020). Long-Term Kidney Function after the Fontan Operation: JACC Review Topic of the Week. J. Am. Coll. Cardiol..

[16] Pundi K., Pundi K.N., Kamath P.S., Cetta F., Li Z., Poterucha J.T., Driscoll D.J., Johnson J.N. (2016). Liver Disease in Patients After the Fontan Operation. Am. J. Cardiol..

[17] Rathgeber S.L., Guttman O.R., Lee A.F., Voss C., Hemphill N.M., Schreiber R.A., Harris K.C. (2020). Fontan-Associated Liver Disease: Spectrum of Disease in Children and Adolescents. J. Am. Heart Assoc..

[18] Asrani S.K., Asrani N.S., Freese D.K., Phillips S.D., Warnes C.A., Heimbach J., Kamath P.S. (2012). Congenital heart disease and the liver. Hepatology.

[19] Rychik J., Atz A.M., Celermajer D.S., Deal B.J., Gatzoulis M.A., Gewillig M.H., Hsia T.Y., Hsu D.T., Kovacs A.H., McCrindle B.W. (2019). Evaluation and Management of the Child and Adult With Fontan Circulation: A Scientific Statement From the American Heart Association. Circulation.

[20] Van De Bruaene A., Hickey E.J., Kovacs A.H., Crean A.M., Wald R.M., Silversides C.K., Redington A.N., Ross H.J., Alba A.C., Billia F. (2018). Phenotype, management and predictors of outcome in a large cohort of adult congenital heart disease patients with heart failure. Int. J. Cardiol..

[21] Engelfriet P., Boersma E., Oechslin E., Tijssen J., Gatzoulis M.A., Thilen U., Kaemmerer H., Moons P., Meijboom F., Popelova J. (2005). The spectrum of adult congenital heart disease in Europe: Morbidity and mortality in a 5 year follow-up period. The Euro Heart Survey on adult congenital heart disease. Eur. Heart J..

[22] Kempny A., Dimopoulos K., Uebing A., Moceri P., Swan L., Gatzoulis M.A., Diller G.P. (2012). Reference values for exercise limitations among adults with congenital heart disease. Relation to activities of daily life--single centre experience and review of published data. Eur. Heart J..

[23] Diller G.P., Giardini A., Dimopoulos K., Gargiulo G., Muller J., Derrick G., Giannakoulas G., Khambadkone S., Lammers A.E., Picchio F.M. (2010). Predictors of morbidity and mortality in contemporary Fontan patients: Results from a multicenter study including cardiopulmonary exercise testing in 321 patients. Eur. Heart J..

[24] Sutherland N., Jones B., d’Udekem Y. (2015). Should We Recommend Exercise after the Fontan Procedure?. Heart Lung Circ..

[25] Khairy P., Poirier N., Mercier L.A. (2007). Univentricular heart. Circulation.

